# Supplementary material for: Ultrafast evolution of bulk, surface and surface resonance states in photoexcited Bi2Te3
Source: Sci Rep. 2021 Mar 1;11:4924. doi: 10.1038/s41598-021-83848-z (PMC7921141; doi:10.1038/s41598-021-83848-z)
Supplement: Supplementary file 1 — Supplementary Information [file 41598_2021_83848_MOESM1_ESM.pdf]

Supplementary Information for Ultrafast Evolution of Bulk, Surface and Surface Resonance states in photoexcited Bi_2Te_3

Hamoon Hedayat[1,2], Davide Bugini[2], Hemian Yi[3], Chaoyu Chen[3], Xingjiang Zhou[3], Giulio Cerullo[2], Claudia Dallera[2], and Ettore Carpene[1]

[1]IFN-CNR Dipartimento di Fisica, Politecnico di Milano, 20133 Milan, Italy
[2]Dipartimento di Fisica, Politecnico di Milano, 20133 Milan, Italy
[3]National Lab for Superconductivity, Institute of Physics, Chinese Academy of Science, 100190 Beijing, China
*ettore.carpene@polimi.it

1. TRARPES GEOMETRY

Fig. 1 schematically illustrates the experimental geometry. The pulses of linearly polarized pump with 1.85 eV photon energy and circularly polarized probe pulses of 6.05 eV were almost collinearly focused on the sample. The sample can be rotated around all three axes of x, y and z (in Fig. 1, θ, β and α, respectively). By changing θ we vary the momentum $k_{||}$. While, by rotating α, we can probe different in-plane crystallographic directions. In Fig. 1, at $\theta = 0$, the time of flight (ToF) analyzer axis is normal to the sample surface while the laser beams impinge at about 45° with respect to the sample surface.

2. CD in Bi_2Te_3

Fig. 2a sketches the out-of-plane spin-OAM structure of Bi_2Te_3 in the surface reciprocal plane and at fixed energy of about 0.2 eV above the Fermi level. The spin-orbit texture of the Dirac-cone follows a threefold symmetry in agreement with Refs.[1–5] In addition, in Fig. 2a the inside circle illustrates the B2 and SR2 nearby the TSS (see main text, Sec. Results and discussion, the photoemission signal of B2 is mixed with SR2). The SR shows an opposite spin direction with respect to the nearest TSS.[6–9] We explored the spin-OAM structure of SR and it reveals a complex spin-OAM configuration following the 3-fold symmetry of TSS spins (as shown in Fig. 2a).

In Fig. 2b, by rotating the sample around the z axis (see Fig. 1), we investigated the CD of TSS, B1 and B2 at different hexagon sides, alternating between $\bar{\Gamma}\bar{K}$ and $\bar{\Gamma}\bar{M}$ directions. We controlled the crystal direction using the in-situ low-energy electron diffraction (LEED). For the rays a1,a2,...,a6 the sample is align with the $\bar{\Gamma}\bar{K}$ direction, for b1,b2 and b3, it is align along $\bar{\Gamma}\bar{M}$. The obtained CD for B1, B2 and TSS as a function of α

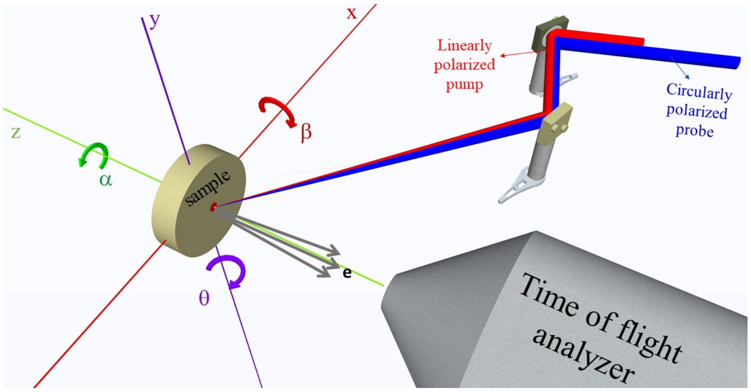

Figure 1. The schematic view of the experimental geometry. Sample can be manipulated in all directions.

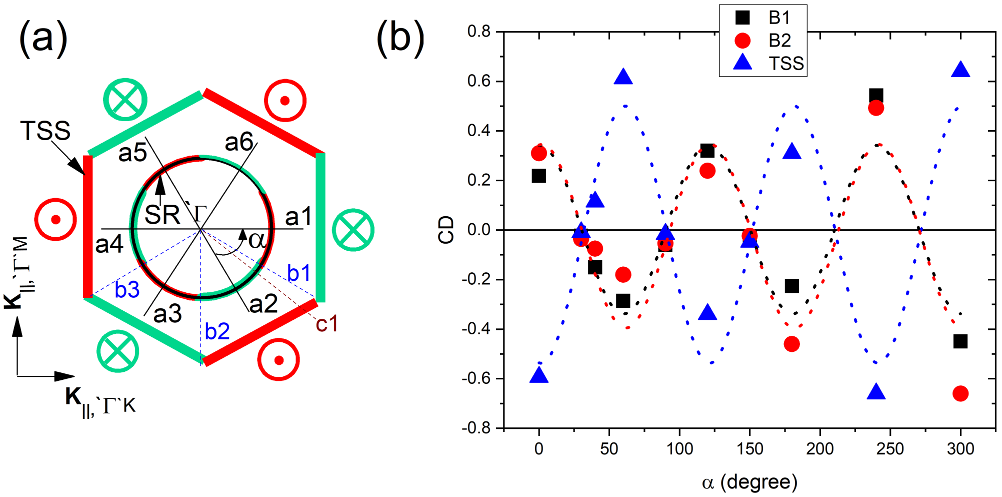

Figure 2. (a) A rough sketch of the out-of-plane spin-OAM structure of TSS and SR in Bi_2Te_3 at about 0.2 eV above the Fermi level. By rotation of α one could explore different crystallographic directions of $k_{||}$. (b) The obtained CD signal for the constant θ ($\simeq 10^\circ$) and β (of the surface be normal to the light propagation plane), and the variable of α as shown in Fig. 2. The sin functions are guide-eyes to follow the 3-fold symmetry of TSS and SRs.

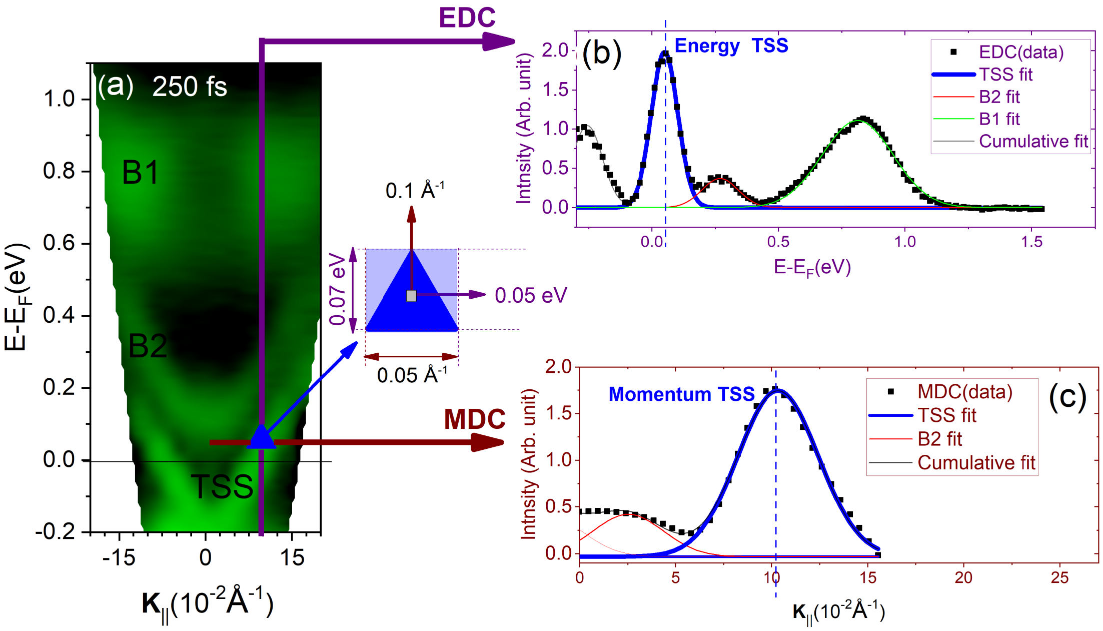

Figure 3. (a) TrARPES map of Bi_2Te_3 along the $\bar{\Gamma}\bar{K}$ for +0.25 ps delay. The blue triangle is a symbol to show the energy-momentum area where the dynamics of $TSS_{[0.05eV]}$ is extracted as depicted in Figure 2 of the main manuscript. Inset shows that the frame, similar to all other frames in Figures 2 and 3 of the main manuscript, has a dimension of $0.05\mathring{A}^{-1} \times 0.07eV$. It is centred at 0.05eV above the Fermi level. Panel (b) shows the energy dispersion curve (EDC) and the fit confirms that the centre of TSS is located at 0.05eV above the Fermi level. Knowing the dispersion of TSS in panel (a) and the energy of TSS (0.05eV) are enough to locate the spot. To demonstrate this, the momentum dispersion curve (MDC) can be extracted as depicted in panel (c). Fitting the MCD by Gaussian functions gives the momentum centre of $TSS_{[0.05eV]}$ area, then here, the centre of the frame is $0.102\mathring{A}^{-1}$.

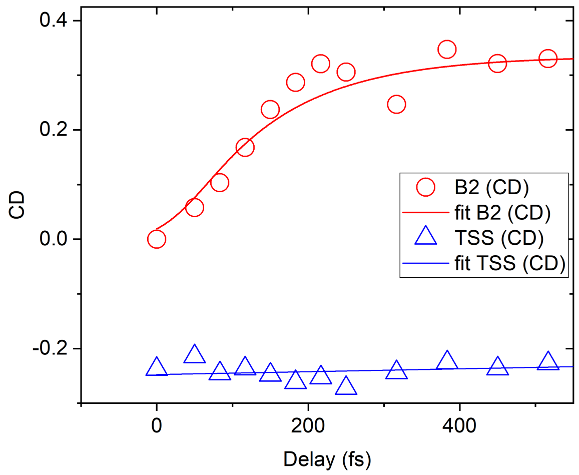

Figure 4. The time dependent CD signal of B2 and TSSs after photoexcitation with s polarized pump pulse. The solid lines are exponential and linear fittings.

is plotted in Fig. 2b. The dashed sine functions are eye-guide to show the three-fold symmetry of CD signals. It is clear that bulk bands and TSS show the opposite CD directions. We attribute the CD behavior of B1 and B2 to the SRs states.

3. THE ENERGY-MOMENTUM AREAS

We define different energy-momentum areas to extract the dynamics of CD and trARPES of TSS, B1 and B2. Since all the bands are symmetric with respect to the $\bar{\Gamma}$ point, the electronic dynamics for each band can be labelled only by the binding energy of the states, $\mathrm{Band}_{[Energy]}$. For example, $\mathrm{TSS}_{[0.05\mathrm{eV}]}$ is the electronic dynamics of TSS extracted for the states at $_{[0.05\mathrm{eV}]}$ above the Fermi level at either side of $\bar{\Gamma}$ point. Here, we explain the energy-momentum areas reported in Figures 2 and 3 of the main manuscript. As an example, Fig. 3 shows the $\mathrm{TSS}_{[0.05\mathrm{eV}]}$ area, the same method was followed for other areas. The dimension of the spot is $(0.05\mathring{A}^{-1} \times 0.07eV)$ (see Inset Fig. 3a). By fitting the energy dispersion curve (EDC) with Gaussian functions, we can derive the energy of each band. Figure. 3b shows that the TSS is located at 0.05eV above the Fermi level. Having the trARPES map (Fig. 3a) and TSS at 0.05eV (Fig. 3b), we can determine the exact location of the area on the map. Fig. 3c shows that the momentum at the centre of the frame will be $0.102\mathring{A}^{-1}$ by fitting the momentum dispersion curve (MDC).

4. PUMP POLARIZATION

To demonstrate that the result presented in the main manuscript (therein Figs 3 and 4) is independent from the pump polarization, we used s-polarized light to excite the system. Fig. 4 show the results. The behavior of CD dynamics perfectly matches the reported data using p-polarized pump as discussed in the main manuscript.

REFERENCES

[1] Fu, L., "Hexagonal warping effects in the surface states of the topological insulator bi 2 te 3," *Physical review letters* **103**(26), 266801 (2009).

[2] Sánchez-Barriga, J., Scholz, M., Golias, E., Rienks, E., Marchenko, D., Varykhalov, A., Yashina, L., and Rader, O., "Anisotropic effect of warping on the lifetime broadening of topological surface states in angle-resolved photoemission from bi 2 te 3," *Physical Review B* **90**(19), 195413 (2014).

[3] Wang, Y., Hsieh, D., Pilon, D., Fu, L., Gardner, D., Lee, Y., and Gedik, N., "Observation of a warped helical spin texture in bi 2 se 3 from circular dichroism angle-resolved photoemission spectroscopy," *Physical Review Letters* **107**(20), 207602 (2011).

[4] Jung, W., Kim, Y., Kim, B., Koh, Y., Kim, C., Matsunami, M., Kimura, S.-i., Arita, M., Shimada, K., Han, J. H., et al., "Warping effects in the band and angular-momentum structures of the topological insulator bi 2 te 3," *Physical Review B* **84**(24), 245435 (2011).

[5] Mirhosseini, H. and Henk, J., "Spin texture and circular dichroism in photoelectron spectroscopy from the topological insulator bi 2 te 3: first-principles photoemission calculations," *Physical review letters* **109**(3), 036803 (2012).

[6] Nurmamat, M., Krasovskii, E., Kuroda, K., Ye, M., Miyamoto, K., Nakatake, M., Okuda, T., Namatame, H., Taniguchi, M., Chulkov, E. V., et al., "Unoccupied topological surface state in bi 2 te 2 se," *Physical Review B* **88**(8), 081301 (2013).

[7] Cacho, C., Crepaldi, A., Battiato, M., Braun, J., Cilento, F., Zacchigna, M., Richter, M., Heckmann, O., Springate, E., Liu, Y., et al., "Momentum-resolved spin dynamics of bulk and surface excited states in the topological insulator bi 2 se 3," *Physical review letters* **114**(9), 097401 (2015).

[8] Jozwiak, C., Sobota, J. A., Gotlieb, K., Kemper, A. F., Rotundu, C. R., Birgeneau, R. J., Hussain, Z., Lee, D.-H., Shen, Z.-X., and Lanzara, A., "Spin-polarized surface resonances accompanying topological surface state formation," *Nature communications* **7** (2016).

[9] Sánchez-Barriga, J., Battiato, M., Krivenkov, M., Golias, E., Varykhalov, A., Romualdi, A., Yashina, L., Minár, J., Kornilov, O., Ebert, H., et al., "Subpicosecond spin dynamics of excited states in the topological insulator bi 2 te 3," *Physical Review B* **95**(12), 125405 (2017).
